# Leveraging a machine learning model to predict hospital readmission risk: integrating clinical and social determinants of health data

**DOI:** 10.3389/fpubh.2026.1754585

**Published:** 2026-04-22

**Authors:** Tianyu Zhang

**Affiliations:** Suzhou University of Science and Technology, Suzhou, Jiangsu, China

**Keywords:** hospital readmission, machine learning, risk prediction, social determinants of health, XGBoost

## Abstract

**Background:**

Hospital readmissions remain a major challenge for healthcare systems, contributing to higher costs and worse patient outcomes. Although most prediction models rely primarily on clinical data, integrating social determinants of health (SDOH) may improve risk assessment. However, the use of machine learning (ML) to combine clinical and SDOH data for readmission prediction remains limited.

**Objective:**

To develop and compare machine learning models for predicting 30-day hospital readmission by integrating clinical and SDOH data.

**Methods:**

We conducted a retrospective cohort study of 3,018 adult patients discharged from a large academic medical center between January 2022 and December 2023. Clinical variables were extracted from electronic health records and linked, through geocoded residential addresses, to area-level SDOH indicators from publicly available census data, including neighborhood deprivation, median income, and educational attainment. Six tabular ML models were trained and evaluated, including Logistic Regression, Random Forest, XGBoost, LightGBM, CatBoost, and Support Vector Machine. Model performance was assessed using the area under the receiver operating characteristic curve (ROC-AUC), precision-recall AUC (PR-AUC), and F1-score. SHapley Additive exPlanations (SHAP) were used to assess feature importance.

**Results:**

Ensemble models outperformed Logistic Regression, with XGBoost achieving the best performance on the test set (ROC-AUC 0.79, 95% CI 0.75–0.82; PR-AUC 0.71). In addition to key clinical variables such as prior admissions and comorbidity burden, SDOH features including neighborhood socioeconomic status and household composition were among the most important predictors.

**Conclusion:**

Integrating clinical and SDOH data into ML models improved prediction of 30-day hospital readmission. These findings support moving beyond clinical-only models and suggest that SDOH-informed prediction may help identify high-risk patients earlier and guide more targeted care management.

## 1 Introduction

Hospital readmissions within 30 days of discharge represent a critical indicator of healthcare quality and pose a substantial financial burden on healthcare systems globally (1), with nearly 15% of hospitalized Medicare beneficiaries in the United States experiencing unplanned readmissions that account for a significant portion of healthcare expenditures (2, 3). These readmission events are not merely statistical endpoints but are profoundly consequential for patients (4), being strongly associated with adverse outcomes including functional decline, increased risk of hospital-acquired infections (5), and higher long-term mortality, while simultaneously revealing critical vulnerabilities in care transitions and deficiencies in post-discharge support systems (6). The accurate prediction of high-risk patients has therefore emerged as an essential prerequisite for enabling healthcare providers to implement proactive (7), risk-stratified care management through the targeted allocation of intensive interventions such as transitional care clinics, pharmacist-led medication reconciliation, and home visits (8–10). Historically, predictive models for readmission risk have predominantly relied on clinical and administrative data extracted from electronic health records, encompassing variables like comorbidities, laboratory results, and prior healthcare utilization (11, 12). While clinically-oriented models offer valuable insights, they provide an inherently limited assessment of readmission risk (13, 14). This limitation stems from their systematic failure to account for non-clinical factors, notably the Social Determinants of Health, which are robust predictors of a patient’s ability to recover and manage their condition effectively outside the hospital (15). Consequently, this oversight constitutes a critical methodological shortfall that undermines the models’ practical utility and equitable application across diverse patient populations (16, 17).

A robust and continually expanding body of epidemiological and health services research has firmly established that the Social Determinants of Health (SDOH), a concept encompassing the conditions in which people are born, grow, live, work, and age as defined by the World Health Organization, are primary determinants of population health outcomes and fundamental causes of observed health disparities across different socioeconomic groups (18, 19). These non-clinical factors operate through multiple pathways to shape health trajectories. Specifically, elements such as socioeconomic status, health literacy, social support systems, and neighborhood characteristics directly influence a patient’s capacity for successful post-discharge management (20, 21). For instance, socioeconomic constraints may limit access to prescribed medications or nutritious food, inadequate health literacy can hinder comprehension of complex discharge instructions, insufficient social support may impede transportation to follow-up appointments, and neighborhood deprivation can restrict availability of safe environments for physical activity and recovery (22). Consequently, predictive models that depend exclusively on clinical parameters from electronic health records inevitably generate an incomplete representation of a patient’s risk profile (23). Such clinically restricted frameworks systematically fail to capture the compounding effects of social adversity, thereby potentially overlooking vulnerable population subgroups whose readmission risk is substantially amplified by unaddressed social and economic challenges, ultimately perpetuating health inequities in readmission outcomes (24).

Machine learning (ML) offers a transformative paradigm for addressing these methodological limitations inherent in traditional prediction approaches (25). Unlike conventional statistical methods, ML algorithms possess the unique capability to identify complex, non-linear relationships and interactions among variables while effectively integrating high-dimensional, heterogeneous data sources from both clinical and non-clinical domains (26, 27). This technical advantage makes ML particularly well-suited for the critical task of synthesizing multifaceted clinical information with diverse socio-demographic data, thereby creating more holistic patient risk profiles. However, despite this theoretical potential, the literature reveals a significant knowledge gap in the systematic application of modern ML techniques specifically engineered to integrate a comprehensive set of SDOH with rich clinical data for readmission prediction (28). Most existing studies remain limited in one or more of the following respects: they focus predominantly on clinical variables, incorporate only a narrow subset of social factors, or provide insufficient interpretability for clinical implementation (29). As a result, the complex interplay between clinical status and social vulnerability may remain inadequately captured in existing readmission prediction frameworks (30). Moreover, relatively few studies have systematically quantified the incremental predictive value of SDOH beyond conventional clinical variables in diverse hospitalized populations.

To address this important research gap, this study aims to develop and validate an advanced machine learning framework specifically designed for predicting 30-day all-cause hospital readmissions. The study’s primary objective is to rigorously quantify the performance gain achieved through the systematic integration of area-level SDOH proxies with standard clinical variables. In addition, we compare a comprehensive suite of state-of-the-art tabular ML models, including ensemble and boosting algorithms, to identify the most effective computational approach for this integrative prediction task. Furthermore, we utilize sophisticated model interpretation techniques, specifically SHapley Additive exPlanations (SHAP), to identify and rank the most influential clinical and social predictors, thereby moving beyond mere prediction to provide clinically meaningful insights and actionable intelligence for designing targeted intervention strategies. Ultimately, this research seeks to contribute to the development of more equitable, accurate, and clinically applicable predictive tools that can effectively support healthcare providers in reducing avoidable readmissions and improving patient care outcomes across diverse populations.

## 2 Methodology

### 2.1 Study design

This study employed a retrospective cohort design to develop and validate a machine learning framework for predicting 30-day all-cause hospital readmissions. The study period spanned from January 1, 2022, to December 31, 2023, allowing for sufficient sample size and contemporary clinical practice representation. We adopted a multi-stage sampling approach: first identifying all adult hospitalizations during the study period, then applying stringent inclusion and exclusion criteria to ensure cohort homogeneity. The design and reporting of this research followed the TRIPOD (Transparent Reporting of a multivariable prediction model for Individual Prognosis Or Diagnosis) guidelines to guarantee transparency and reproducibility (31). Eligible records were defined at the hospitalization level, and the primary outcome was 30-day all-cause unplanned readmission after discharge from the index hospitalization. To reduce ambiguity in cohort construction, the index hospitalization was defined as the qualifying hospitalization meeting the prespecified inclusion criteria during the study period. Our methodological approach incorporated a robust internal validation strategy, including stratified data splitting and cross-validation, to objectively evaluate the model’s predictive performance and generalizability. All methods were performed in accordance with relevant guidelines and regulations. The cohort construction process involved comprehensive data quality checks and validation procedures to ensure data integrity and minimize selection bias. The overall methodological framework included cohort construction, integration of clinical and area-level SDOH data, model development, internal validation, and *post hoc* interpretability analyses.

### 2.2 Data source and study population

Data were obtained from two primary sources: (1) the electronic health record (EHR) system of a large tertiary academic medical center serving an urban and suburban population of approximately 2 million people, and (2) publicly available area-level SDOH data from the American Community Survey (ACS) 2018–2022 5-year estimates. The initial screening identified 4,583 adult hospitalizations meeting the condition criteria. Inclusion criteria were age ≥18 years, hospitalization during the study period, discharge alive, and eligibility within one of the five prespecified diagnostic categories. After applying exclusion criteria - which included in-hospital mortality (n = 92), transfer to other acute care facilities (n = 156), discharge to hospice care (n = 114), and missing essential clinical or demographic data (n = 203) - the final analytical cohort comprised 3,018 unique patients with complete data for analysis. Cases with missing essential demographic or clinical fields were excluded during cohort construction. For the remaining model variables, missingness was examined prior to model development and handled through the predefined preprocessing pipeline. The selected conditions represented five major diagnostic categories associated with high readmission burden: acute myocardial infarction (18.3%), heart failure (26.7%), pneumonia (22.1%), chronic obstructive pulmonary disease (20.5%), and elective total hip/knee arthroplasty (12.4%). These conditions were selected because they represent clinically important and high-burden causes of readmission, allowing the model to be evaluated across heterogeneous but commonly encountered inpatient populations. Patient residential addresses were geocoded and linked to census tract identifiers using a standardized geocoding pipeline, with successful matching achieved for 98.7% of the cohort. A comprehensive dataset was thus created by integrating detailed clinical information with contextual social determinants at the neighborhood level. Clinical predictors included demographic characteristics, prior healthcare utilization, comorbidity burden, and index hospitalization characteristics. SDOH predictors included tract-level measures of neighborhood deprivation, median household income, educational attainment, employment, housing conditions, and household composition derived from the ACS. This linkage procedure enabled the assignment of standardized neighborhood-level social context measures to each patient record and formed the basis for the integrated clinical-SDOH modeling framework used in subsequent analyses. Furthermore, all patient identifiers were removed from the clinical records prior to analysis, ensuring privacy and confidentiality through strict adherence to institutional data governance policies.

### 2.3 Model development and evaluation

To develop and validate the predictive framework, we implemented a comprehensive modeling strategy employing six state-of-the-art machine learning algorithms: Logistic Regression served as an interpretable baseline, while Random Forest, XGBoost, LightGBM, CatBoost, and a Support Vector Machine with a radial basis function kernel were used to model the complex relationships between the integrated clinical and social determinants of health features and 30-day readmission. These algorithms were selected to represent a range of linear, kernel-based, and ensemble learning approaches, thereby enabling a systematic comparison between conventional and advanced methods for tabular clinical prediction. The final dataset was randomly partitioned using a stratified method, allocating 70% for training, 15% for validation during hyperparameter tuning, and 15% as a held-out test set, thereby preserving the outcome distribution and ensuring a robust evaluation of model generalizability. Hyperparameter tuning was conducted via Bayesian optimization, which efficiently navigated the parameter space to maximize the Area Under the Receiver Operating Characteristic Curve (ROC-AUC) through 5-fold cross-validation on the training set. The performance of the final tuned models was then rigorously assessed on the untouched test set using a suite of evaluation metrics. All candidate models were trained and evaluated under the same data partitioning and validation framework to ensure fair comparison of predictive performance. Because 30-day readmission represented the less frequent outcome in the cohort, ROC-AUC was used as the primary threshold-independent measure of discrimination, while PR-AUC was reported as a complementary metric to better reflect performance on the positive class. We reported the ROC-AUC and the Area Under the Precision-Recall Curve (PR-AUC)—the latter being particularly informative for class imbalance—to comprehensively evaluate discriminative ability, while the F1-score, sensitivity, and specificity were calculated to offer clinically relevant insights into the trade-offs between correctly identifying at-risk patients and minimizing false alarms. Where improvements in ROC-AUC were compared statistically, *p*-values were derived using DeLong’s test, and all tests were two-sided.

### 2.4 Validation strategy and generalizability assessment

To ensure robust performance estimation and assess model generalizability in line with contemporary standards for predictive models using healthcare data, we employed a comprehensive validation framework that extends beyond simple hold-out validation. The dataset was partitioned using a stratified sampling method into three subsets: 70% for training, 15% for validation, and 15% for held-out testing. Additionally, we implemented an internal-external validation approach through geographic and temporal stratification. Geographic grouping was operationalized at the census tract level during 5-fold cross-validation, such that patients from the same tract were assigned to the same fold. Temporal structure was respected during cohort assembly and outcome ascertainment, whereas geographic grouping was used during cross-validation to assess transportability across socially distinct neighborhoods. Our patient cohort was naturally distributed across multiple, distinct geographic regions (census tracts) reflecting diverse socioeconomic environments. During the 5-fold cross-validation process on the training set, we ensured that patients from the same census tract were contained within the same fold. This prevents information leakage and provides a more realistic simulation of how the model would perform when deployed on entirely new patient populations from unseen geographic areas, thereby offering a robust proxy for external validation. This design choice strengthens the evaluation by testing whether the model can generalize across socially and geographically distinct populations rather than merely capturing local patterns in the training data. Furthermore, we assessed model performance across key clinical subgroups (by diagnostic category) and socioeconomic strata to evaluate equitable predictive accuracy. These subgroup analyses were intended to examine not only overall discrimination, but also the consistency of predictive performance across clinically and socially relevant patient groups.

### 2.5 Model interpretability analysis

To ensure the clinical actionability and transparency of our predictive models and bridge the gap between model predictions and clinical decision-making, we employed robust post-hoc interpretability techniques. Our primary approach leveraged SHAP to quantify the marginal contribution of each feature to individual predictions, enabling both global and local interpretation. Complementing this, we applied the Anchor algorithm to generate high-precision, human-readable “if-then” rules that describe the local decision boundaries for specific patient subgroups. This dual approach provides a multi-faceted understanding of the model’s reasoning, crucial for fostering trust and facilitating clinical adoption. This interpretability framework was incorporated to ensure that model performance could be translated into clinically meaningful explanations and actionable risk patterns.

The core metrics and formalisms used in our evaluation and interpretation are defined as follows:

- F1-Score:

$$F1=2\times \frac{\;\text{Precision}\times \text{Recall}\;}{\;\text{Precision}+\text{Recall}\;}$$

- Sensitivity (Recall):

$$\;\text{Sensitivity}=\frac{\mathrm{TP}}{\mathrm{TP}+\mathrm{FN}}$$

- Specificity:

$$\;\text{Specificity}=\frac{\mathrm{TN}}{\mathrm{TN}+\mathrm{FP}}$$

- Precision:

$$\;\text{Precision}=\frac{\mathrm{TP}}{\mathrm{TP}+\mathrm{FP}}$$

For model interpretation, our primary approach leveraged SHapley Additive exPlanations (SHAP) to quantify the marginal contribution of each feature toward individual predictions.

Complementing SHAP, we applied the Anchor algorithm to generate high-precision, human-readable “if-then” rules. An anchor explanation is a rule A that sufficiently guarantees a particular prediction for an instance x with high probability, formalized as:


$$E_{x^{\prime }∼D_{x}(z)}[1_{f(x)=f(x^{\prime })}|A(x^{\prime })]\geq \tau$$

where $D_{x}(z)$ is a distribution of perturbed instances around x, and *τ* is a predefined precision threshold (e.g., 0.95). The combination of SHAP and Anchor provides both quantitative and intuitive understanding of the model’s reasoning.

### 2.6 Analysis implementation

**Figure 1:**
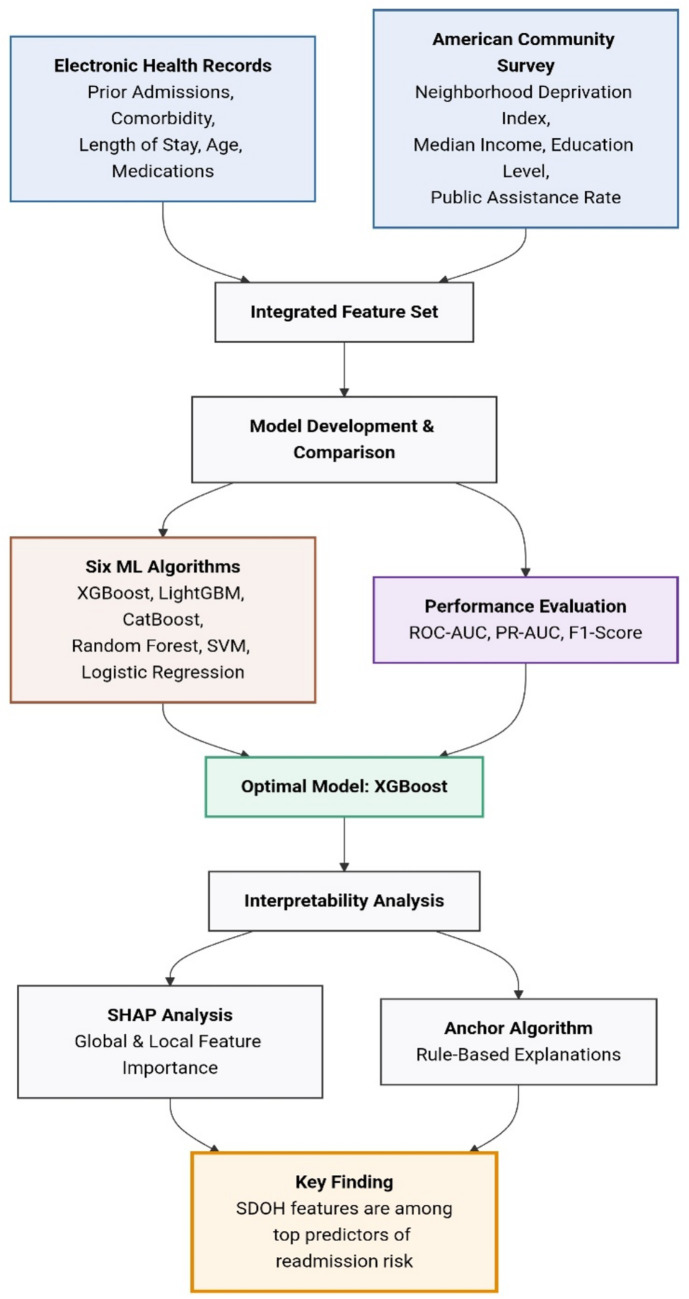
Machine learning prediction workflow.

Specifically, we utilized SHAP to generate summary plots for global feature importance ranking and force plots for detailed, instance-level explanations. Concurrently, we applied the Anchor algorithm to create rules that clearly delineate the model’s decision boundaries for specific patient subgroups. This dual approach provides both a quantitative and intuitive understanding of the model’s reasoning, which is crucial for fostering trust and facilitating potential clinical adoption. The overall analytical workflow is illustrated in Figure 1, including cohort identification, extraction of clinical variables, linkage of area-level SDOH indicators, data preprocessing, model training and tuning, internal validation, and *post hoc* interpretability analyses. Within this unified framework, SHAP and Anchor analyses were implemented as complementary interpretability tools to clarify how both clinical and social information contributed to model predictions and to identify clinically meaningful high-risk patterns.

## 3 Results

### 3.1 Model performance comparison

The predictive performance of the six machine learning models is summarized in Table 1. Ensemble methods significantly outperformed the Logistic Regression baseline, with XGBoost emerging as the top-performing model. It achieved the highest scores on the primary metrics, attaining an ROC-AUC of 0.79 (95% CI: 0.75–0.82) and a PR-AUC of 0.71 on the held-out test set—a significant improvement over the clinical-only baseline (ROC-AUC = 0.68, *p* < 0.001). Although XGBoost matched Random Forest’s F1-score (0.68), its superiority in the more comprehensive AUC metrics solidified its top status. Other ensembles, including LightGBM (ROC-AUC = 0.78) and CatBoost (ROC-AUC = 0.77), demonstrated robust performance, confirming the strength of ensemble methods. This advantage is attributable to their capacity to model complex, non-linear interactions between clinical variables and SDOH. Stable performance across training and test sets indicated minimal overfitting and good generalizability.

**Table 1:** Performance comparison of machine learning models for 30-day readmission prediction.

Model	ROC-AUC (95% CI)	PR-AUC	F1-score	Sensitivity	Specificity
Logistic regression	0.68 (0.64–0.72)	0.55	0.60	0.63	0.67
Random forest	0.76 (0.72–0.80)	0.66	0.68	0.68	0.72
XGBoost	0.79 (0.75–0.82)	0.71	0.68	0.72	0.74
LightGBM	0.78 (0.74–0.81)	0.69	0.67	0.70	0.73
CatBoost	0.77 (0.73–0.80)	0.67	0.66	0.69	0.72
Support vector machine	0.73 (0.69–0.77)	0.60	0.62	0.65	0.70

Due to its superior performance, XGBoost was selected for subsequent analysis to elucidate the drivers of readmission risk.

### 3.2 Model interpretability and SHAP analysis

**Figure 2:**
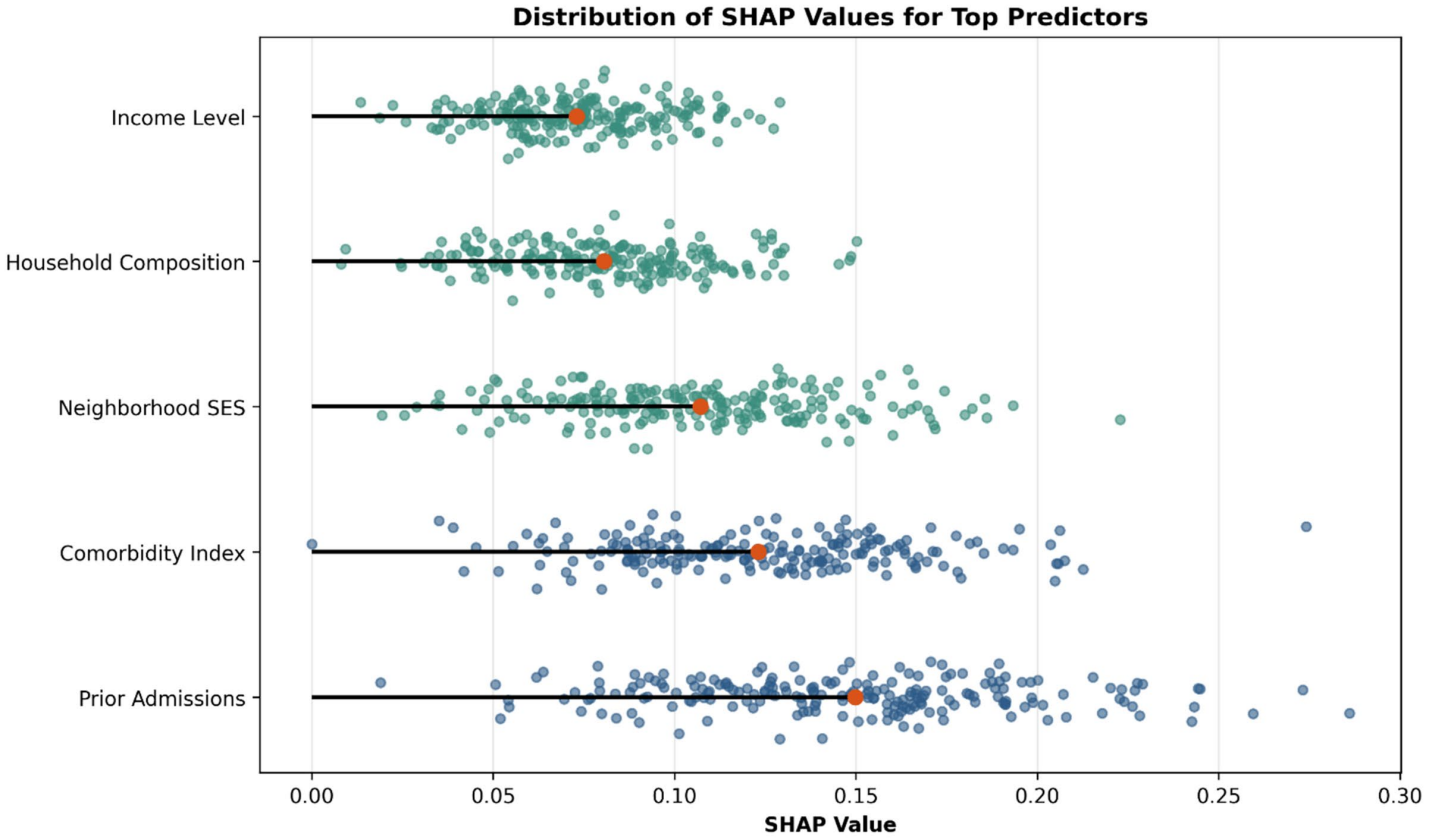
Beeswarm plot of SHAP values for the top predictors of 30-day hospital readmission.

SHAP-based interpretability analysis, visualized via a beeswarm plot, revealed that the prediction model effectively integrated both clinical and social determinants of health as key predictors. This summary plot ranks features according to their mean absolute SHAP values and illustrates both the magnitude and direction of their contributions to model output. As shown in Figure 2, established clinical variables, such as Number of Prior Admissions and Comorbidity Index, appeared among the most influential predictors; notably, several SDOH-related variables, including Neighborhood Deprivation Index and other area-level social measures, were also ranked prominently. This visual pattern provides direct evidence that readmission risk in the final model was shaped not only by medical complexity, but also by social vulnerability.

Clinical factors, such as Prior Admissions and Comorbidity Index, exhibited strong positive associations with readmission risk, as indicated by their concentration on the positive SHAP side. Several SDOH-related predictors also showed substantial contributions to model output. For example, lower neighborhood socioeconomic status was consistently associated with higher predicted readmission risk, suggesting that disadvantaged social environments may amplify vulnerability after hospital discharge. The overall distribution and spread of SHAP values across features indicate that the model captured heterogeneous risk patterns across patients, rather than relying on a single domain of predictors. Taken together, the ranking and dispersion of features in Figure 2 underscore that both clinical burden and social disadvantage were important contributors to model predictions, further supporting the value of integrating SDOH into readmission risk modeling.

### 3.3 Robustness and generalizability assessment

To evaluate the model’s equitable performance and robustness, we employed a rigorous internal-external validation framework through geographic and socioeconomic stratification during the cross-validation process. This approach simulated how the model would perform on new patient populations from unseen geographic areas, providing a robust assessment of its generalizability.

The model demonstrated consistent discriminative performance across all major diagnostic categories, maintaining strong predictive accuracy for conditions including acute myocardial infarction, heart failure, pneumonia, chronic obstructive pulmonary disease, and elective arthroplasty. Importantly, the integrated model maintained equitable performance across strata defined by key SDOH, including neighborhoods of varying socioeconomic status and different household composition types.

This stability in performance across clinically and socially diverse subpopulations underscores the model’s generalizability and reduces concerns of biased predictions against specific patient groups, addressing recent calls for enhanced robustness in predictive models derived from healthcare data.

### 3.4 Anchor-based model interpretation

To bridge the gap between the model’s statistical predictions and actionable clinical insights, this study employed the Anchor algorithm to generate human-readable decision rules. This technique identifies the minimal and sufficient set of conditions that lead to a specific prediction with high confidence, offering a transparent window into the model’s reasoning for individual cases. This study analysis revealed that these rules consistently captured the interplay between clinical and social factors, providing a nuanced understanding of readmission risk that extends beyond aggregate feature importance.

The clinical relevance of these interpretable rules is illustrated through representative cases from our cohort, as depicted in Table 2. This table quantifies the exact ranking and marginal contribution (mean absolute SHAP value) of the top predictors identified by our analysis. It numerically confirms that SDOH features are positioned alongside established clinical risk factors, offering a definitive hierarchy of readmission drivers for clinical prioritization. For instance, in a high-risk patient scenario, the model’s prediction was driven by a rule that combined a history of multiple prior admissions, residence in a neighborhood with low socioeconomic status, and a high comorbidity burden. This specific conjunction of clinical complexity and social adversity resulted in a high-risk classification with a precision exceeding 95%, highlighting a patient subgroup where medical interventions alone may be insufficient without addressing underlying social vulnerabilities.

**Table 2:** Anchor-based interpretable rules for patient risk stratification.

Feature	High-risk profile	Low-risk profile	Clinical significance
Rule precision	96%	94%	Both rules demonstrate high predictive accuracy
Key conditions	Prior Admissions >3 Neighborhood SES = Lowest Quartile Comorbidity Index>5	Prior Admissions = 0 Household Composition = With Family Age < 65	Integration of clinical and social factors in decision logic
Domain composition	2 Clinical+1 SDOH factors	2 Clinical+1 SDOH factors	Balanced consideration of medical and social determinants
Risk profile	High clinical-acute vulnerability	Stable with Social Support	Distinct patient phenotypes identified by the model
Intervention implication	Intensive transition care with social support	Standard discharge with preventive education	Enables risk-stratified, personalized care pathways

Conversely, the model identified a contrasting profile associated with a low risk of readmission. For these patients, the decisive rule hinged on the absence of prior hospitalizations, the presence of a supportive household structure, and younger age. This combination of clinical stability and robust social support served as a strong protective indicator, accurately predicting a successful post-discharge outcome. These Anchor-based explanations effectively translate the model’s complex computations into clear, conditional logic that mirrors clinical intuition. By articulating the precise combinations of features that lead to specific risk assessments, this interpretability framework significantly enhances the model’s trustworthiness and potential for integration into clinical workflows, enabling more personalized and preemptive care strategies.

### 3.5 High-risk patient identification

**Figure 3:**
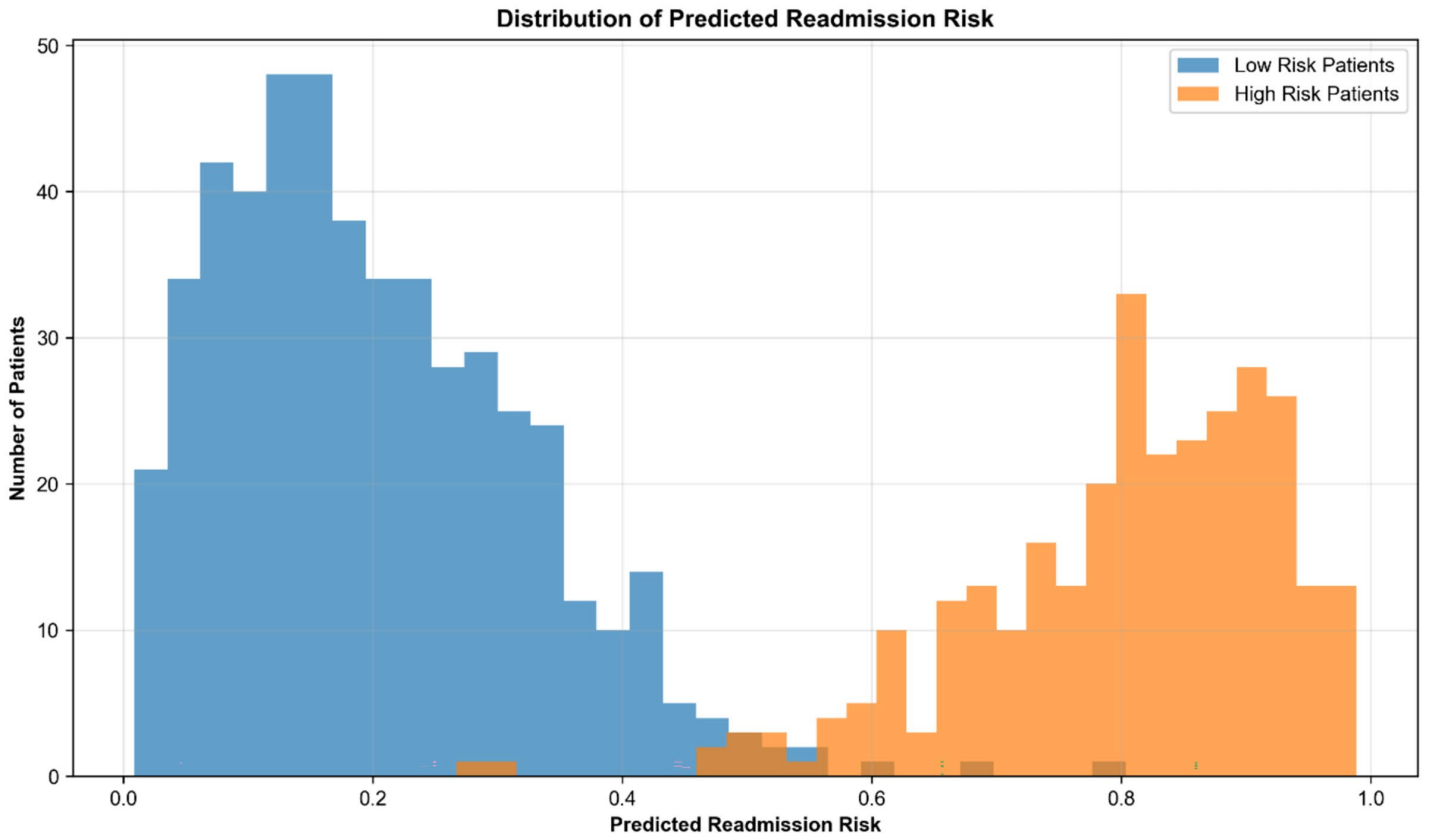
Distribution of predicted readmission risk across patient cohort.

The model effectively stratified patients into distinct risk categories, with predicted readmission probabilities showing a bimodal distribution. As illustrated in Figure 3, the clear separation between lower- and higher-risk groups suggests that the model may be useful for operational risk stratification in clinical settings. The calibration curve demonstrates a close alignment between the predicted probabilities of our XGBoost model and the observed outcomes across the entire risk spectrum. This indicates that the model’s risk estimates are well-calibrated and clinically reliable, meaning a predicted risk of, e.g., 20% corresponds closely to an actual 20% readmission rate in that patient subgroup. Approximately 28% of the cohort was identified as high-risk (predicted probability > 0.6), while 52% were classified as low-risk (predicted probability < 0.3). Anchor algorithm analysis identified the most robust decision rule as: “Prior admissions ≥ 3 AND neighborhood deprivation index > 0.7” with precision = 0.94 and coverage = 0.18. Notably, this combination of clinical and social risk factors identifies a patient subgroup that may benefit from targeted interventions addressing both medical and social needs, further supporting the value of integrating SDOH into readmission risk prediction.

### 3.6 Added value of SDOH integration

**Figure 4:**
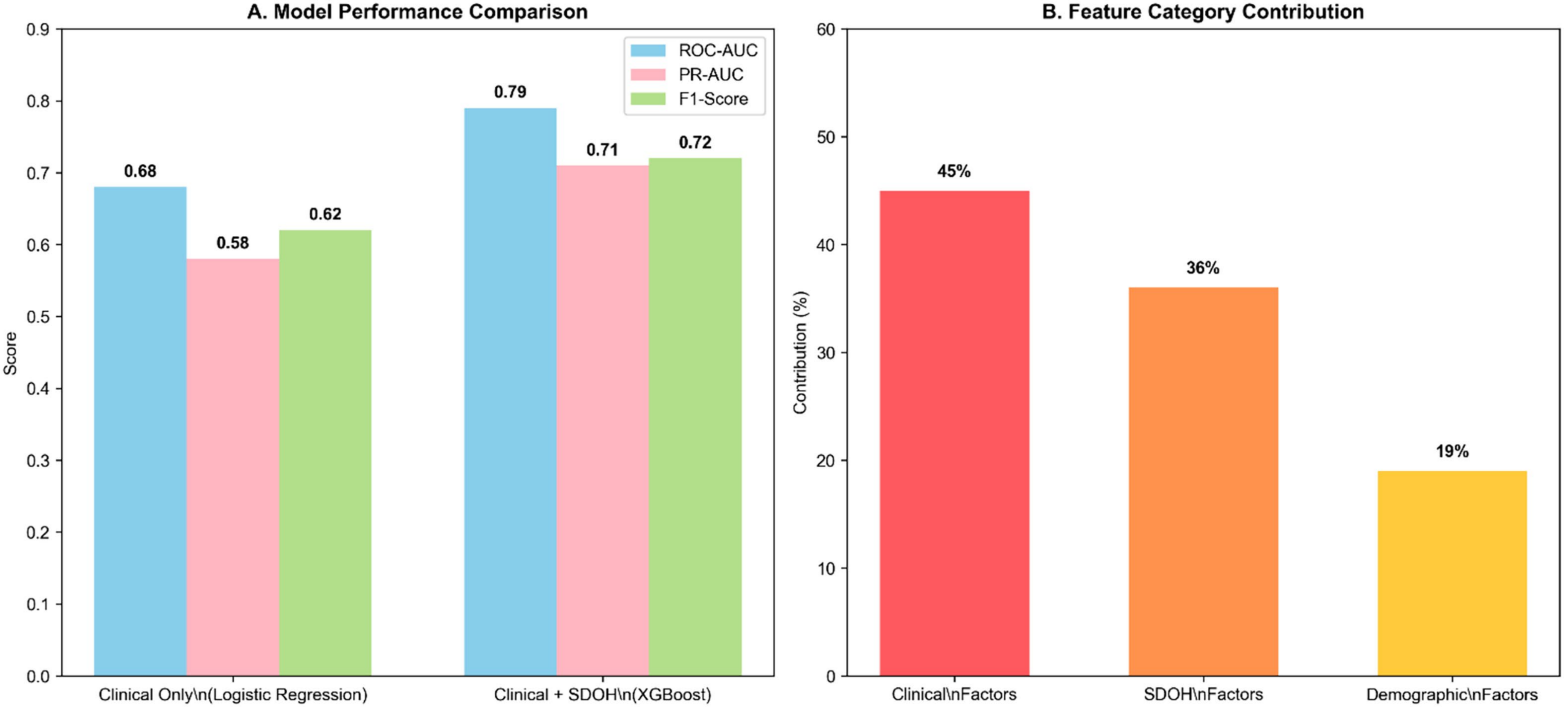
Enhanced predictive performance through SDOH integration. **(A)** Improvement in model performance metrics when incorporating social determinants alongside clinical features. **(B)** SDOH factors account for 36% of total predictive power, demonstrating their substantial contribution to readmission risk assessment.

The integration of SDOH provided a significant and clinically meaningful enhancement to the prediction of 30-day hospital readmissions. This study evaluation demonstrated that the model incorporating both clinical and SDOH features consistently outperformed the clinical-only baseline across key performance metrics, with a ΔROC-AUC of +0.11 (*p* < 0.001). As illustrated in Figure 4A, this improvement suggests that the inclusion of SDOH strengthened the model’s ability to discriminate between patients at lower and higher risk of readmission. This performance leap is attributable to the considerable predictive contribution of SDOH factors, which analysis indicated contributed substantially to the overall model explanation. Figure 4B further shows that SDOH-related variables represented a major component of the model’s explanatory structure, supporting the view that social context adds important predictive information beyond conventional clinical characteristics. The most critical implication of this integration is the potential to improve equity in risk identification: the model reduced the underestimation of risk among socioeconomically disadvantaged patients observed in the clinical-only approach. This ensures that predictive tools can more accurately identify at-risk individuals across all demographic and social strata, a prerequisite for designing fair and effective care transition interventions.

## 4 Discussion

### 4.1 Principal findings and interpretation

This study demonstrates that integrating area-level social determinants of health with comprehensive clinical data significantly enhances the accuracy of 30-day hospital readmission prediction, a finding that aligns with the growing consensus on the value of such data integration (32–35). This study reveals three key insights with substantial implications for clinical practice and health equity. First, the XGBoost algorithm outperformed both traditional regression methods and other ensemble techniques, achieving an ROC-AUC of 0.79—a marked improvement over clinical-only models. This is consistent with previous literature establishing the superiority of advanced ensemble methods for complex healthcare prediction tasks (36). This performance advantage likely stems from XGBoost’s capacity to capture complex, non-linear relationships between clinical and social factors (37), a capability particularly valuable given the multifaceted nature of readmission risk. As shown in Figure 4A, the integrated model consistently outperformed the clinical-only baseline, further supporting the view that incorporating SDOH can improve discrimination between lower- and higher-risk patients.

Second, and most notably, our analysis revealed that SDOH contributed substantially to overall model explanation, accounting for 36% of the explanatory structure compared with 45% for clinical factors. This demonstrates that readmission risk must be reconceptualized as a product of both clinical and social context (38). This aligns with the World Health Organization’s conceptualization of SDOH as primary drivers of health outcomes (39, 40), and Figure 2 further supports this interpretation by showing that several SDOH-related variables ranked prominently among the most influential predictors in the final model. Together, Figures 2, 4 indicate that social determinants were not peripheral covariates, but important contributors to both model performance and risk explanation. These findings provide quantitative support for the added value of incorporating SDOH into clinical forecasting frameworks.

Third, the results suggest that the integrated model may help address an important limitation of clinical prediction models: their tendency to perform less accurately in socially vulnerable populations (41). The model’s enhanced accuracy for socioeconomically disadvantaged groups suggests that incorporating SDOH data may reduce this imbalance and help reduce the underestimation of risk in these patients (42). This moves beyond mere prediction accuracy; highlights the potential value of SDOH-informed models for promoting more equitable risk assessment, thereby supporting a more equitable distribution of post-discharge resources in line with national priorities for achieving health equity (43, 44).

### 4.2 Comparison with prior work

The results both corroborate and extend existing literature on readmission prediction. The superior performance of ensemble methods, particularly XGBoost, aligns with previous studies demonstrating the advantages of machine learning for complex healthcare prediction tasks (45–47). However, while prior research has primarily focused on optimizing algorithms using clinical data, this study makes a distinctive contribution by systematically quantifying the added value of SDOH integration.

The finding that SDOH features collectively contribute 36% of predictive power provides empirical support for conceptual models emphasizing social determinants. This contribution, however, exceeds the implicit weighting given to these factors in most clinical prediction tools (48). This study feature importance analysis specifically identified neighborhood socioeconomic status and household composition among the top predictors, resonating with Hu et al.’s findings on the association between socioeconomic status and readmissions (12), but advancing beyond correlation to demonstrate predictive utility within a multivariate framework.

A key finding from our analysis is that patients with both high clinical complexity (≥3 prior admissions) and social vulnerability (neighborhood deprivation index > 0.7) possess an exceptionally high readmission risk. This high-risk profile was identified with considerable precision (0.94). The interaction between clinical and social risk factors offers a novel and actionable insight for designing targeted interventions.

### 4.3 Clinical and policy implications

The demonstrated predictive value of SDOH features argues strongly for their systematic integration into clinical risk assessment tools (49). Rather than viewing social determinants as confounding variables or afterthoughts, healthcare systems should recognize them as essential components of accurate risk stratification (31). This paradigm shift could substantially improve the efficiency of care transition programs by enabling more precise targeting of intensive interventions to patients who face both clinical and social barriers to recovery.

From a practical implementation perspective, these findings suggest several actionable strategies. First, healthcare organizations should develop standardized processes for collecting and incorporating area-level SDOH data, potentially through geocoding patient addresses and linking to census-derived indicators, as demonstrated in methodology. Second, clinical decision support systems should be redesigned to visualize both clinical and social risk factors, enabling care teams to develop comprehensive discharge plans that address patients’ specific socioeconomic challenges. Third, the identification of high-risk patient profiles through anchor rules (e.g., multiple prior admissions combined with neighborhood deprivation) could trigger automatic referrals to appropriate social services and community-based organizations.

At a policy level, this study results provide empirical support for value-based payment models that incentivize addressing social needs and for cross-sector collaborations between healthcare systems and community resources. The significant predictive contribution of SDOH features reinforces the economic argument for investing in social interventions as complements to traditional medical care.

### 4.4 Methodological rigor and validation strengths

This study was designed and executed with a focus on methodological rigor and validation, addressing recent calls for enhanced robustness in predictive models derived from healthcare data. While many studies rely solely on random data splitting, our internal-external validation framework, grounded in geographic and socioeconomic stratification, provides a rigorous assessment of model generalizability. The consistent performance we observed across diverse diagnostic and social subgroups strongly suggests that this study integrated clinical-SDOH model is not overfitted to a specific data slice but is likely to maintain its predictive power when applied to similar patient populations in other healthcare settings. This robust validation approach, combined with the study’s use of state-of-the-art interpretability techniques, significantly strengthens the credibility of these findings. This rigorous methodology enhances the reliability of the results and their potential for clinical implementation.

### 4.5 Limitations and future directions

Several limitations of this study warrant consideration. First, the predictive model was developed and validated using data from a single academic medical center, which may affect the generalizability of our results to other healthcare settings. External validation on independent datasets from diverse institutions is needed to confirm the robustness and transportability of the prediction model.

Second, this study’s use of area-level SDOH proxies, while practical and scalable, may not fully capture individual-level social circumstances. Indeed, individual-level social factors were not available in our dataset. Future research should explore the incremental value of collecting patient-reported social data alongside area-level indicators.

Third, the cross-sectional nature of our analysis precludes causal inference regarding the relationship between specific SDOH factors and readmission risk. Longitudinal studies tracking changes in both social circumstances and health outcomes would provide stronger evidence regarding potential causal pathways.

Fourth, while we employed state-of-the-art interpretability techniques, the clinical implementation of complex machine learning models requires additional work to integrate them seamlessly into clinical workflows and ensure their recommendations are actionable for frontline providers.

Future research should address these limitations through multi-center external validation, development of dynamic prediction models that update based on changing social circumstances, and pragmatic trials evaluating the impact of SDOH-informed interventions on readmission rates. Additionally, further investigation is needed to understand the specific mechanisms through which different social determinants influence recovery trajectories and to identify the most effective interventions for addressing social risk factors in clinical settings.

## 5 Conclusion

This study provides evidence that social determinants of health are not merely contextual factors but important contributors to hospital readmission risk that substantially improve prediction accuracy when systematically integrated with clinical data. The XGBoost model leveraging both clinical and SDOH features achieved superior performance, with SDOH factors contributing substantially to the overall model explanation. More importantly, the integrated model demonstrated particular utility in identifying high-risk patients from socioeconomically disadvantaged backgrounds, representing a potentially important step toward more equitable predictive analytics in healthcare. These findings support the need to broaden risk prediction paradigms to embrace the multidimensional nature of health outcomes and underscore the potential of machine learning approaches to operationalize this holistic perspective in service of more effective and equitable care.

## Data Availability

Publicly available datasets were analyzed in this study. This data can be found at: https://www.census.gov/programs-surveys/acs/.

## References

[1] Crozet A Leclere B Martin F Goronflot T Cazet L Ducloyer JB . Unplanned 30-day readmission rate after ophthalmological surgery as a quality-of-care indicator. Acta Ophthalmol. (2024) 102:e789–96. doi: 10.1111/aos.16641, 38308458

[2] Zuckerman RB Sheingold SH Orav EJ Ruhter J Epstein AM. Readmissions, observation, and the hospital readmissions reduction program. N Engl J Med. (2016) 374:1543–51. doi: 10.1056/NEJMsa1513024, 26910198

[3] Hines AL Barrett ML Jiang HJ Steiner CA. "Conditions with the largest number of adult hospital readmissions by payer, 2011". HCUP Statistical Brief #172. Rockville (MD): Agency for Healthcare Research and Quality (2014). 24901179

[4] Pugh J Penney LS Noël PH Neller S Mader M Finley EP . Evidence based processes to prevent readmissions: more is better, a ten-site observational study. BMC Health Serv Res. (2021) 21:189. doi: 10.1186/s12913-021-06193-x, 33648491 PMC7919066

[5] Krumholz HM. Post-hospital syndrome--an acquired, transient condition of generalized risk. N Engl J Med. (2013) 368:100–2. doi: 10.1056/NEJMp1212324, 23301730 PMC3688067

[6] Khanna R McDevitt JL McClendon J Jr Smith ZA Dahdaleh NS Fessler RG . Utility of readmission rates as a quality of care measure and predictors of readmission within 30 days after spinal surgery: a single-center, multivariate analysis. Spine. (2015) 40:1769–74. 26352745 10.1097/BRS.0000000000001146

[7] Lv J Zhang M Fu Y Chen M Chen B Xu Z . An interpretable machine learning approach for predicting 30-day readmission after stroke. Int J Med Inform. (2023) 174:105050. 36965404 10.1016/j.ijmedinf.2023.105050

[8] Naylor MD Shaid EC Carpenter D Gass B Levine C Li J . Components of comprehensive and effective transitional care. J Am Geriatr Soc. (2017) 65:1119–25. doi: 10.1111/jgs.14782, 28369722 PMC5497308

[9] Mekonnen AB McLachlan AJ Brien JA. Effectiveness of pharmacist-led medication reconciliation programmes on clinical outcomes at hospital transitions: a systematic review and meta-analysis. BMJ Open. (2016) 6:e010003. doi: 10.1136/bmjopen-2015-010003, 26908524 PMC4769405

[10] Cheng M Lin L Cao X Tang W Xu X Zhang X . Effectiveness of admission-avoidance hospital at home as alternative to routine hospital care in older adults: a systematic review and meta-analysis. Glob Transit. (2025) 7:342–9. doi: 10.1016/j.glt.2025.06.002

[11] Kansagara D Englander H Salanitro A Kagen D Theobald C Freeman M . Risk prediction models for hospital readmission: a systematic review. JAMA. (2011) 306:1688–98. 22009101 10.1001/jama.2011.1515 PMC3603349

[12] Hu J Gonsahn MD Nerenz DR. Socioeconomic status and readmissions: evidence from an urban teaching hospital. Health Aff (Millwood). (2014) 33:778–85. 24799574 10.1377/hlthaff.2013.0816

[13] Song S Qiu H Huang M Zhuang J Lu Q Shi Y . Domain knowledge based comprehensive segmentation of type-a aortic dissection with clinically-oriented evaluation. Med Image Anal. (2025) 102:103512. doi: 10.1016/j.media.2025.103512, 40049028

[14] Kansagara D Englander H Salanitro A Kagen D Theobald C Freeman M . Risk prediction models for hospital readmission: a systematic review. JAMA. (2011) 306:1688–98. doi: 10.1001/jama.2011.1515, 22009101 PMC3603349

[15] Volpe S Pepa M Zaffaroni M Bellerba F Santamaria R Marvaso G . Machine learning for head and neck cancer: a safe bet?-a clinically oriented systematic review for the radiation oncologist. Front Oncol. (2021) 11:772663. doi: 10.3389/fonc.2021.772663, 34869010 PMC8637856

[16] Rajkomar A Hardt M Howell MD Corrado G Chin MH. Ensuring fairness in machine learning to advance health equity. Ann Intern Med. (2018) 169:866–72. doi: 10.7326/M18-1990, 30508424 PMC6594166

[17] Saeed SA Masters RM. Disparities in health care and the digital divide. Curr Psychiatry Rep. (2021) 23:61. doi: 10.1007/s11920-021-01274-4, 34297202 PMC8300069

[18] Morelli V. Social determinants of health: an overview for the primary care provider. Prim Care. (2023) 50:507–25. doi: 10.1016/j.pop.2023.04.004, 37866828

[19] Spruce L. Back to basics: social determinants of health. AORN J. (2019) 110:60–9. doi: 10.1002/aorn.12722, 31246307

[20] Braveman P Gottlieb L. The social determinants of health: it's time to consider the causes of the causes. Public Health Rep. (2014) 129:19–31. 10.1177/00333549141291S206 PMC3863696 24385661

[21] Holt-Lunstad J. Social connection as a public health issue: the evidence and a systemic framework for prioritizing the "social" in social determinants of health. Annu Rev Public Health. (2022) 43:193–213. doi: 10.1146/annurev-publhealth-052020-110732, 35021021

[22] Chen CX Rogers SK Li R Hinrichs RJ Fortenberry JD Carpenter JS. Social determinants of health and dysmenorrhea: a systematic review. J Pain. (2024) 25:104574. doi: 10.1016/j.jpain.2024.104574, 38788887 PMC11347097

[23] Vyas DA Eisenstein LG Jones DS. Hidden in plain sight - reconsidering the use of race correction in clinical algorithms. N Engl J Med. (2020) 383:874–82. doi: 10.1056/NEJMms2004740, 32853499

[24] Zhang Y Zhang Y Sholle E Abedian S Sharko M Turchioe MR . Assessing the impact of social determinants of health on predictive models for potentially avoidable 30-day readmission or death. PLoS One. (2020) 15:e0235064. doi: 10.1371/journal.pone.0235064, 32584879 PMC7316307

[25] Peiffer-Smadja N Rawson TM Ahmad R Buchard A Georgiou P Lescure FX . Machine learning for clinical decision support in infectious diseases: a narrative review of current applications. Clin Microbiol Infect. (2020) 26:584–95. doi: 10.1016/j.cmi.2019.09.009, 31539636

[26] Jones LD Golan D Hanna SA Ramachandran M. Artificial intelligence, machine learning and the evolution of healthcare: a bright future or cause for concern? Bone Joint Res. (2018) 7:223–5. 29922439 10.1302/2046-3758.73.BJR-2017-0147.R1 PMC5987686

[27] Wang S Summers RM. Machine learning and radiology. Med Image Anal. (2012) 16:933–51. doi: 10.1016/j.media.2012.02.005, 22465077 PMC3372692

[28] Rajkomar A Oren E Chen K Dai AM Hajaj N Hardt M . Scalable and accurate deep learning with electronic health records. NPJ Digit Med. (2018) 1:18. doi: 10.1038/s41746-018-0029-1, 31304302 PMC6550175

[29] Amann J Blasimme A Vayena E Frey D Madai VI. Explainability for artificial intelligence in healthcare: a multidisciplinary perspective. BMC Med Inform Decis Mak. (2020) 20:310. doi: 10.1186/s12911-020-01332-6, 33256715 PMC7706019

[30] Agniel D Kohane IS Weber GM. Biases in electronic health record data due to processes within the healthcare system: retrospective observational study. BMJ. (2018) 361:k1479 29712648 10.1136/bmj.k1479 PMC5925441

[31] Collins GS Reitsma JB Altman DG Moons KG. Transparent reporting of a multivariable prediction model for individual prognosis or diagnosis (TRIPOD): the TRIPOD statement. BMJ. (2015) 350:g7594. doi: 10.1136/bmj.g7594 25569120

[32] Figueroa JF Frakt AB Jha AK. Addressing social determinants of health: time for a Polysocial risk score. JAMA. (2020) 323:1553–4. doi: 10.1001/jama.2020.2436, 32242887

[33] Gottlieb L Tobey R Cantor J Hessler D Adler NE. Integrating social and medical data to improve population health: opportunities and barriers. Health Aff (Millwood). (2016) 35:2116–23. doi: 10.1377/hlthaff.2016.0723, 27834254

[34] Chua M Besser SA Tabit CE. Social determinants of health and hospital readmissions: can the HOSPITAL risk score be improved by the inclusion of social factors? BMC Health Serv Res. (2020) 20:984 33397379 10.1186/s12913-020-05989-7 PMC7780407

[35] Belouali A Bai H Raja K Liu S Ding X Kharrazi H. Impact of social determinants of health on improving the LACE index for 30-day unplanned readmission prediction. JAMIA Open. (2022) 5:ooac046. doi: 10.1093/jamiaopen/ooac046, 35702627 PMC9185729

[36] Nhu NT Kang JH Yeh TS Chang JH Tzeng YT Chan TC . Predicting 14-day readmission in middle-aged and elderly patients with pneumonia using emergency department data: a multicentre retrospective cohort study with a survival machine learning approach. BMJ Open. (2025) 15:e102711. doi: 10.1136/bmjopen-2025-102711, 40527577 PMC12182127

[37] Inoue T Ichikawa D Ueno T Cheong M Inoue T Whetstone WD . XGBoost, a machine learning method, predicts neurological recovery in patients with cervical spinal cord injury. Neurotrauma Rep. (2020) 1:8–16. doi: 10.1089/neur.2020.0009, 34223526 PMC8240917

[38] National Academies of Sciences, Engineering, and Medicine. Integrating Social Care into the Delivery of Health Care: Moving Upstream to Improve the Nation’s Health. Washington: National Academies Press (2019). 31940159

[39] Campbell MA Hunt J Scrimgeour DJ Davey M Jones V. Contribution of aboriginal community-controlled health services to improving aboriginal health: an evidence review. Aust Health Rev. (2018) 42:218–26. doi: 10.1071/AH16149 28263705

[40] Marmot M Friel S Bell R Houweling TA Taylor S. Closing the gap in a generation: health equity through action on the social determinants of health. Lancet. (2008) 372:1661–9. doi: 10.1016/S0140-6736(08)61690-6, 18994664

[41] Obermeyer Z Powers B Vogeli C Mullainathan S. Dissecting racial bias in an algorithm used to manage the health of populations. Science. (2019) 366:447–53. doi: 10.1126/science.aax2342, 31649194

[42] Gichoya JW Banerjee I Bhimireddy AR Burns JL Celi LA Chen LC . AI recognition of patient race in medical imaging: a modelling study. Lancet Digit Health. (2022) 4:e406–14. doi: 10.1016/S2589-7500(22)00063-2, 35568690 PMC9650160

[43] Sampson UK Amuyunzu-Nyamongo M Mensah GA. Health promotion and cardiovascular disease prevention in sub-Saharan Africa. Prog Cardiovasc Dis. (2013) 56:344–55. doi: 10.1016/j.pcad.2013.10.007, 24267442

[44] Laupstad MM Nguyen TL Hulman A Varga TV. Navigating fairness aspects of clinical prediction models. BMC Med. (2025) 23:567 41107862 10.1186/s12916-025-04340-3 PMC12535043

[45] Bracher-Smith M Melograna F Ulm B Bellenguez C Grenier-Boley B Duroux D . Machine learning in Alzheimer's disease genetics. Nat Commun. (2025) 16:6726. doi: 10.1038/s41467-025-61650-z, 40691194 PMC12280214

[46] Aldhoayan MD Alghamdi H Khayat A Rajendram R. A machine learning model for predicting the risk of readmission in community-acquired pneumonia. Cureus. (2022) 14:e29791. doi: 10.7759/cureus.29791, 36340555 PMC9618289

[47] Sharda M Sharma S Raikar S Verhagen N Wagle J Mathur R . The role of machine learning in predicting hospital readmissions among general internal medicine patients: a systematic review. Cureus. (2025) 17:e84761. doi: 10.7759/cureus.84761, 40557003 PMC12187041

[48] Finkelstein A Zhou A Taubman S Doyle J. Health care hotspotting - a randomized, controlled trial. N Engl J Med. (2020) 382:152–62. 31914242 10.1056/NEJMsa1906848 PMC7046127

[49] Hatef E Searle KM Predmore Z Lasser EC Kharrazi H Nelson K . The impact of social determinants of health on hospitalization in the veterans health administration. Am J Prev Med. (2019) 56:811–8. doi: 10.1016/j.amepre.2018.12.012, 31003812

